# Eliminating interference by anthocyanin in chlorophyll estimation of sweet potato (Ipomoea batatas L.) leaves

**DOI:** 10.1186/1999-3110-55-11

**Published:** 2014-01-30

**Authors:** Wen-Dar Huang, Kuan-Hung Lin, Ming-Huang Hsu, Meng-Yuan Huang, Zhi-Wei Yang, Pi-Yu Chao, Chi-Ming Yang

**Affiliations:** 1 Department of Agronomy, National Taiwan University, Daan, Taipei ROC 106 Taiwan; 2 Graduate Institute of Biotechnology, Chinese Culture University, Xulin, Taipei ROC 111 Taiwan; 3 Refining and Manufacturing Research Institute, CPC Corporation, Taiwan, Chiayi ROC 600 Taiwan; 4 Biodiversity Research Center, Academia Sinica, Nangang, Taipei ROC 115 Taiwan; 5 Taoyuan District Agricultural Research and Extension Station, Council of Agriculture, Taoyuan, ROC 327 Taiwan; 6 Department of Food, Health and Nutrition Science, Chinese Culture University, Xulin, Taipei ROC 111 Taiwan

**Keywords:** Sweet potato leaves, Chlorophyll, Anthocyanins, Spectral reflectance

## Abstract

**Background:**

Spectral reflectance was evaluated for its usefulness as a nondestructive estimation of chlorophyll (Chl) content from three cultivars of sweet potato (*Ipomoea batatas* L.) with green, yellow, and purple leaves grown in a greenhouse for 22 days. While the green and yellow leaves contain variant amount of photosynthetic pigments without or with little level of anthocyanins, the purple leaves, except large amount of photosynthetic pigments, have high quantity of anthocyanins.

**Results:**

For green and yellow leaves, the reciprocal reflectance (R^-1^) and derived indices incorporating near infrared (NIR) reflectance, [(R_λ_)^-1^ - (R_NIR_)^-1^] and [(R_NIR_/R_λ_) - 1], in the green and red edge spectral ranges were shown to be strongly correlated (*r*^2^ = 0.8 ~ 0.9) with the chlorophyll content. The root mean square error (RMSE) of the chlorophyll content estimation using these indices was < 50 mg m^-2^. However, when purple leaves containing high levels of anthocyanins were included in the sample, R^-1^ in the green spectral range and the above-mentioned indices displayed much weaker correlations with the chlorophyll content. The RMSE of chlorophyll estimation using these indices in the green spectral range sharply increased to > 110 mg m^-2^ when the sample included purple leaves. The new index, [1 - (R_λ_/R_NIR_)], was therefore inferred and developed to eliminate the distorting effect of anthocyanins on chlorophyll content estimation using reflectance in the green spectral range. For leaves with high levels of anthocyanins, the correlation between [1 - (R_λ_/R_NIR_)] and the chlorophyll content remained strong (r^2^ = 0.8 ~ 0.9) in the green spectral range, and the RMSE was minimal.

**Conclusion:**

The reflectance index, [1 - (R_λ_/R_NIR_)], therefore represents a new and useful parameter for estimating leaf chlorophyll content in leaves with any level of anthocyanins such as purple rice leaf.

**Electronic supplementary material:**

The online version of this article (doi:10.1186/1999-3110-55-11) contains supplementary material, which is available to authorized users.

## Multilingual abstracts

Please see Additional file 1 for translations of the abstract into the six official working languages of the United Nations.

## Background

The chlorophylls (Chls), Chl-*a* and -*b*, are the most important plant pigments as they are necessary for photosynthesis. Chlorophylls absorb solar radiation and convert light energy to chemical energy. The amount of solar radiation that a leaf absorbs is determined mainly by chlorophylls. Thus, the foliar chlorophyll content directly affects the photosynthetic potential and primary production (Filella *et al*., 1995). Since chlorophyll contains much of the nitrogen content of a leaf, the chlorophyll content can provide an indirect estimation of a plant’s nutrient status (Moran *et al*., 2000). The chlorophyll content changes throughout the different stages of plant development, and it universally decreases in situations of plant stress and during senescence (Merzlyak and Gitelson, 1995).

Traditional methods of measuring leaf chlorophyll content are destructive. They require leaf extraction with organic solvents and spectrophotometric or HPLC measurements in solution, and are considered time-consuming and expensive (Gitelson *et al*., 2006; Blackburn, 2007). However, much attention has been devoted to developing nondestructive leaf optical methods as alternative means to measure leaf chlorophyll contents. These methods include transmittance and reflectance spectroscopy for chlorophyll content measurement, and can be applied across spatial scales because of their simplicity, rapidity, and nondestructive nature (Chappelle *et al*., 1992; Buschmann and Nagel 1993; Main *et al*., 2011).

Many theoretical models based on leaf reflectance were developed to predict the leaf chlorophyll content, water content, and other variables associated with the vegetative structure (Inoue *et al*., 1993; Jacquemoud *et al*., 1996; Dawson *et al*., 1998). Relationships between reflectance in the visible region of the spectrum and the chlorophyll content are fundamentally nonlinear (Gitelson and Merzlyak, 1997). However, reciprocal reflectances (R^-1^) in the green and red edge spectral regions were found to be closely related to the chlorophyll content in leaves with a wide range of pigment compositions and contents (Gitelson and Merzlyak, 1994). Indices using these spectral bands were suggested for chlorophyll estimation in leaves of various plant species (Zou *et al*., 2011; Dillen *et al*., 2012; Huang *et al*., 2012). Gitelson et al. (2003) reported that the index, (R_λ_)^-1^ - (R_NIR_)^-1^, was linearly proportional to the chlorophyll content in the wide spectral bands 525 ~ 555 and 695 ~ 725 nm. Moreover, a derived index incorporating near infrared (NIR) reflectance, [(R_λ_)^-1^ - (R_NIR_)^-1^] × R_NIR_, was also used to adjust for differences in leaf structure, and this algorithm gives highly accurate estimation of chlorophyll levels. Recently, a three-band conceptual model of remotely estimating total chlorophyll contents in leaves was developed and produced accurate estimation (Gitelson *et al*., 2006).

Leaf colors are determined by pigments, and anthocyanins are responsible for the red coloration in plant tissues (Ji *et al*., 1992; Gould *et al*., 1995). Therefore, leaves containing anthocyanins tend to be red or purple. Light in the spectral band at around 550 nm is absorbed by anthocyanins. The development of nondestructive methods for chlorophyll estimation using the green spectral band is more difficult for leaves containing anthocyanins, because their main absorption band is also located in the green range (., Merzlyak *et al* 2003). In anthocyanin-free leaves, nondestructive chlorophyll estimation are possible using either the green band (around 550 nm), red edge band (around 700 nm), or an NIR band of > 750 nm. For leaves containing anthocyanins, use of only the red edge band or an NIR band at > 750 nm is recommended (Gitelson *et al*., 2006).

In applying the above developed models for nondestructively estimating chlorophyll contents in leaves with or without anthocyanins (Gitelson *et al*., 2006), big error was found in anthocyanin-containing leaves, suggesting that anthocyanins may affect the nondestructive estimation of chlorophyll content in all purple leaves. Actually, the same phenomenon was also found and bothered the authors for more than fifteen years in biochemical and destructive measurement of chlorophyll in leaves with any level of anthocyanins; in short, the higher content the anthocyanins, the bigger error the chlorophyll measurement (personal data not published). We referred that the anthocyanins strongly absorbing around 500–550 nm may strongly affect the chlorophyll-related compounds, including all chlorophyll biosynthesis and degradation intermediates, strongly absorbing 400–475 nm and 550–700 nm. The more concentration of anthocyanins lead to the more distortion effect on chlorophyll measurement.

Therefore, the objectives of this study were to investigate the properties of the reflectance spectra of sweet potato leaves with and without anthocyanins, and to develop techniques for nondestructive chlorophyll estimation in leaves with a wide range of pigment contents. A technique was developed to eliminate the effects of anthocyanin on reflectance in the green spectral range on chlorophyll estimation, and a new index for estimating chlorophyll contents is finally proposed.

## Methods

### Plant materials and culture practice

Three leafy vegetable sweet potato [*Ipomoea batatas* (L.) Lam] cultivars of Taoyuan 2, green, yellow, and purple were used in this study. The green leaf cultivar was selected by the Taoyuan District Agricultural Research and Extension Station (TDARES) for food consumption of its leaves. Yellow and purple cultivars are local varieties from Taoyuan, and contain high amounts of carotenoids. Terminals about 40 cm in length were taken from sturdy vines, and cultivated in plastic boxes 60 cm long, 22 cm wide, and 15 cm deep, that contained a medium of sand, vermiculite, and loamy soil in a volume ratio of 2:1:1. Specimens were planted in a greenhouse at the TDARES, and evenly spaced at intervals of 60 cm to encourage similar growth rates and sizes. Plants were treated with the nutrients N:P_2_O_5_:K_2_O (0.1:0.2:0.3 g/box) weekly, watered every other day to maintain optimal irrigation, and allowed to grow for 22 d before the following measurements of spectral reflectance were made.

### Measurement of spectral reflectance

Adaxial reflectance spectra of leaves were recorded at a rate of 600 nm/min using an U-3010 spectrophotometer (Hitachi, Tokyo, Japan) equipped with an integrating sphere attachment. The diffuse reflectance of the leaves was measured at values of 400 ~ 800 nm (R_400_ ~ R_800_) with a spectral resolution of 1 nm and against barium sulfate as a reference standard. The reflectance was expressed as a ratio of the radiance of the leaf to the radiance of the reference. The spectra were recorded for sections of leaves between the main veins.

### Pigment determinations

Chlorophylls and carotenoids were isolated from leaves by homogenization in liquid nitrogen and subsequent threefold extraction with 80% acetone (v/v). After centrifugation for 5 min at 1500 × *g*, the absorbance of the supernatant was measured at 663.6, 646.6, and 440.5 nm (Porra et al., 1989). Leaf samples were also extracted with 1% (w/v) HCl in methanol, and the anthocyanin contents were assayed spectrophotometrically. The relative amounts of anthocyanins were expressed by [A_530_ - 0.333A_657_] m^-2^ (Mancinelli *et al*., 1975). Absorbance was measured with a Hitachi U-2000 UV-visible spectrophotometer.

### Statistical analysis

To develop model datasets, 48 leaves from the three varieties were used. Validation was done using independent datasets derived from 24 leaves, and the chlorophyll content was calculated using reflectance data from the model’s validation datasets. The predicted chlorophyll content was compared to chlorophyll levels physically measured, and the root mean square error (RMSE) of the predictions was calculated. Relationships between the reflectance indices and chlorophyll contents were examined using simple linear regression models. All statistical analyses were conducted using JMP software, vers. 5.01 (SAS Institute, Cary, NC, USA).

## Results

### Pigment contents

Variations in pigment contents of green, yellow, and purple leaves were observed and are given in Table 1. The chlorophyll content varied from 99 mg m^-2^ in yellow leaves to 615 mg m^-2^ in purple leaves. Purple leaves had the highest chlorophyll content, green leaves had an intermediate level, and yellow leaves had the lowest amount. The mean chlorophyll content in yellow (132 mg m^-2^) leaves was about 2 ~ 3-times lower than that of green (408 mg m^-2^) and purple (478 mg m^-2^) leaves. While green and yellow leaves were found to be free of anthocyanins, purple leaves contained much of them.

**Table 1:** **The content of chlorophylls (Chl), carotenoids, and anthocyanins in leaves of three varieties of sweet potato**

Data set	***n***	Chl (mg m^-2^)		Carotenoids (mg m^-2^)	Anthocyanins (A_530_-0.333A_657_) m^-2^
		Total	Mean		
Model development					
Green	16	319 ~ 515	408	74 ~ 88	0
Yellow	16	99 ~ 182	132	36 ~ 51	0
Purple	16	336 ~ 615	478	73 ~ 88	0.43 ~ 2.90
Model validation					
Green	8	237 ~ 431		68 ~ 83	0
Yellow	8	104 ~ 145		33 ~ 47	0
Purple	8	297 ~ 545		74 ~ 89	0.35 ~ 2.71

*n* is the number of leaves in each data set.

### Reciprocal reflectance

**Figure 1:**
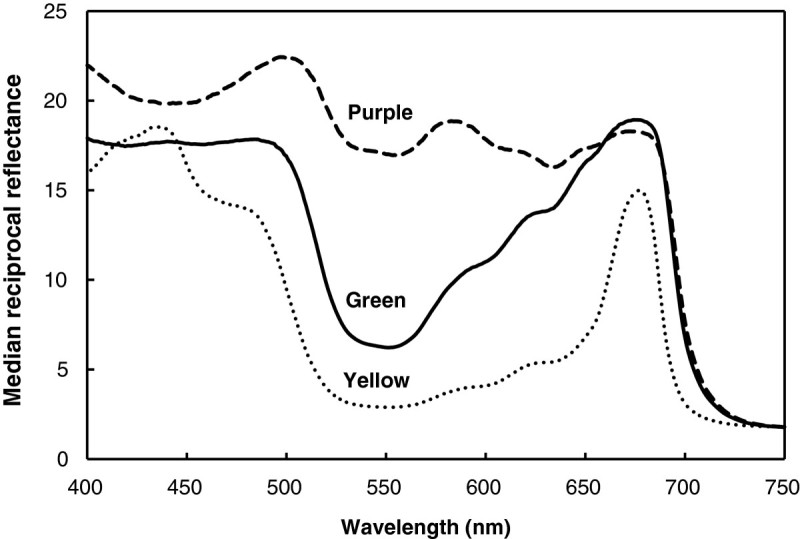
**Reciprocal reflectance spectra for leaves of three varieties of sweet potato.**

The reciprocal reflectance spectra (R^-1^) in the blue wavelength ranging 400 ~ 500 nm was highest in purple leaves, but lower in green and yellow leaves (Figure 1). Values of R^-1^ were affected by absorption by both chlorophyll and carotenoids in this range. There were differences in R^-1^ values of green and yellow leaves in the 500 ~ 700-nm range, with yellow leaves being lower. A blue-shift accompanied a decrease in chlorophyll content at both the green edge (around 530 nm) and red edge (around 700 nm). In purple leaves, the change in R^-1^ values was not notable in this range. Purple leaves, which had the highest chlorophyll content, also had the highest R^-1^; conversely yellow leaves, with the lowest chlorophyll content, also had the lowest R^-1^ (Table 1, Figure 1).

### Correlations of reflectance indices with the chlorophyll content, and the RMSE with chlorophyll estimates

**Figure 2:**
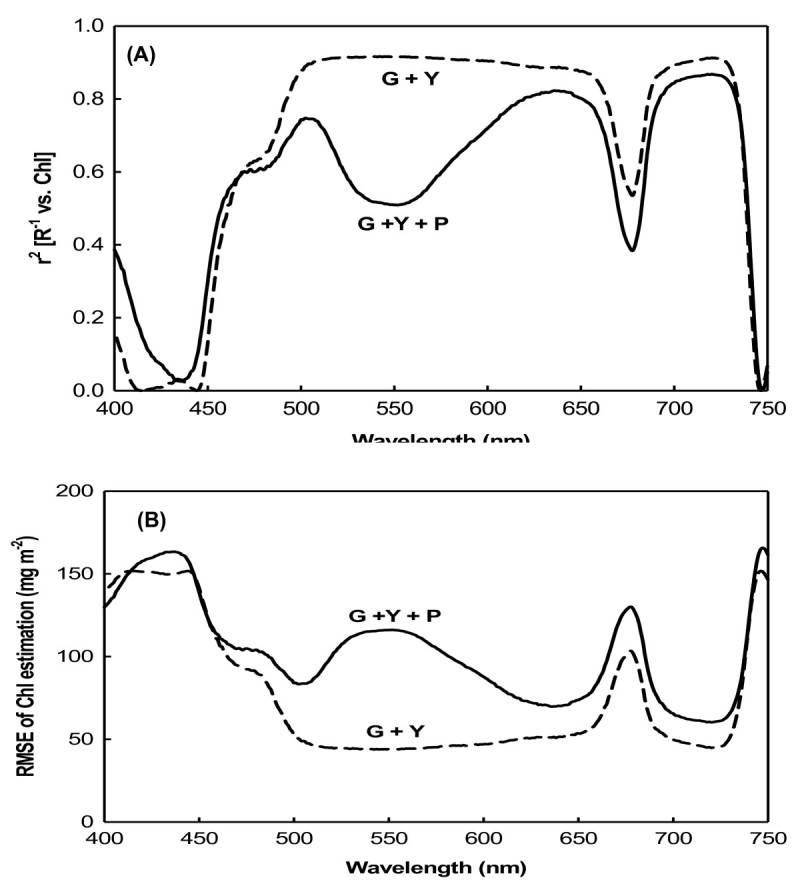
**Coefficient between reciprocal reflectance and chlorophyll content and its RMSE. (A)** Coefficient between reciprocal reflectance (R^-1^) and chlorophyll content; **(B)** root mean square error (RMSE) of the chlorophyll estimation by R^-1^. G+Y, green and yellow leaves only; G+Y+P: green, yellow, and purple leaves.

To investigate spectral bands where R^-1^ was sensitive to the chlorophyll content, coefficients (*r*^2^) of R^-1^ with the chlorophyll content and the RMSE with reflectance estimates of the chlorophyll content were examined. The model development data were divided into two sets. Because we focused on the anthocyanin contents of purple leaves, one set included only green and yellow leaves, while the other included purple leaves. For green and yellow (G + Y) leaves, *r*^2^ was minimal in the blue (400 ~ 500 nm) and red (around 680 nm) ranges. A consistently high level of correlation (*r*^2^ = 0.8 ~ 0.9) was observed over a broad range of 500 ~ 650 nm (green-red region of the spectrum) and near 700 nm (red edge) (Figure 2A). Compared to the other set, the set including purple leaves (G + Y + P) had a significantly lower *r*^2^ (of 0.51) between R^-1^ and the chlorophyll content in the green region of the spectrum at 500 ~ 600 nm (Figure 2A). In addition, the correlation was strong for all leaves (G + Y + P) in the red region at around 640 nm and at the red edge at around 700 nm. This demonstrates the applicability of R^-1^ as a method of measuring the total chlorophyll content. In these spectral ranges, the RMSE for chlorophyll estimation was minimal. The RMSE was < 50 mg m^-2^ for the green and yellow (G + Y) set, and > 70 mg m^-2^ for the set with all (G + Y + P) leaves (Figure 2B).

**Figure 3:**
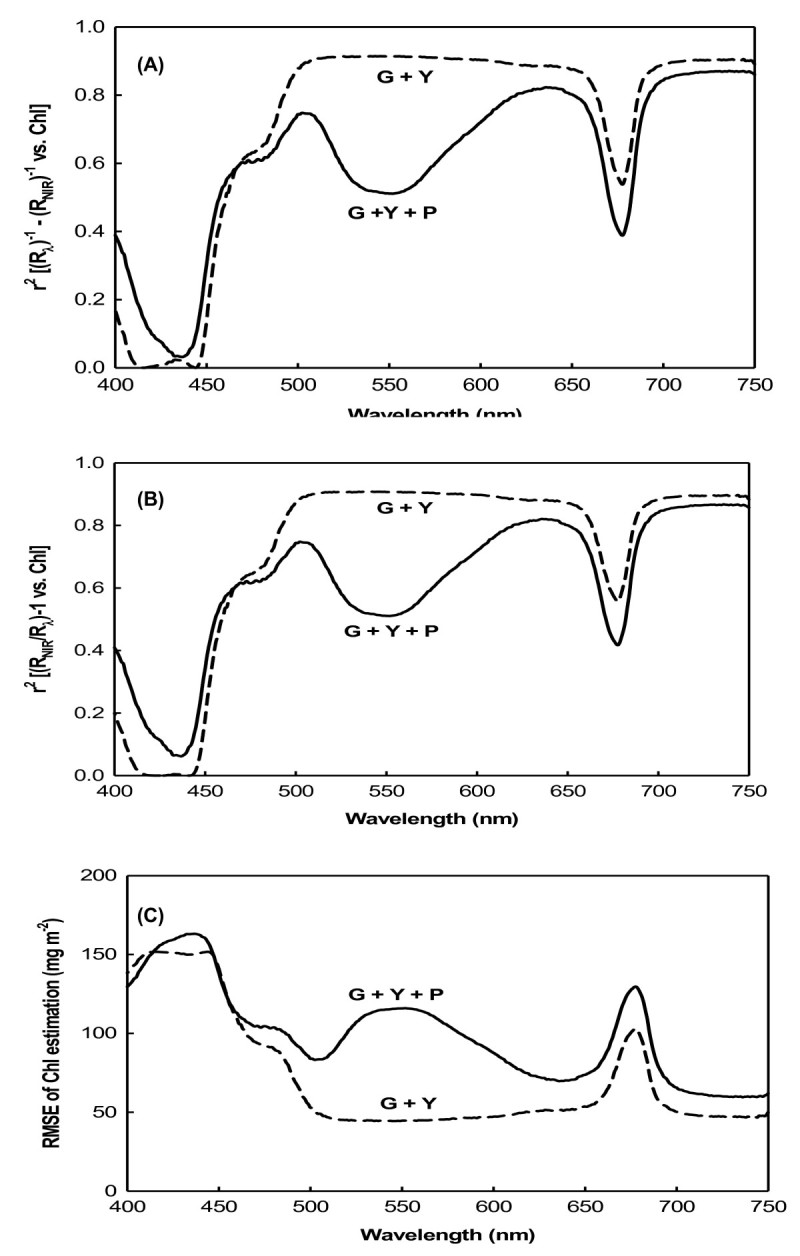
**Coefficients between [(R**_**λ**_**)**^**-1**^**- (R**_**NIR**_**)**^**-1**^**] and [(R**_**NIR**_**/ R**_**λ**_**) - 1] and the chlorophyll content and their RMSE. (A)** Coefficient between [(R_λ_)^-1^ - (R_NIR_)^-1^] and the chlorophyll content; **(B)** coefficient between [(R_NIR_/R_λ_) - 1] and the chlorophyll content; **(C)** root mean square error (RMSE) of chlorophyll estimation by R^-1^.

Two indices derived from the reciprocal reflectance, [(R_λ_)^-1^ - (R_NIR_)^-1^] and [(R_NIR_/R_λ_) - 1], were correlated to the total chlorophyll content in wide spectral ranges. Coefficients of [(R_λ_)^-1^ - (R_NIR_)^-1^] vs. chlorophyll (Figure 3A) and [(R_NIR_/R_λ_) - 1] vs. chlorophyll (Figure 3B) presented a broad flat maximum in the respective spectral ranges of 500 ~ 650 and 700 ~ 740 nm for green and yellow (G + Y) leaves, while R_NIR_ is the reflectance in the NIR range of 750 ~ 800 nm. In these spectral ranges for G + Y leaves, the RMSE of the chlorophyll estimation by these indices was < 50 mg m^-2^ (Figure 3C). When using all leaves, including purple (G + Y + P), as the dataset, *r*^2^ decreased down to <0.6 in the green region of the spectrum at 500 ~ 600 nm (Figure 3A, B), but the RMSE increased to > 110 mg m^-2^ (Figure 3C). It is obvious that anthocyanins affect the chlorophyll estimation, and it seems that no existed models can eliminate this distortion effect.

**Figure 4:**
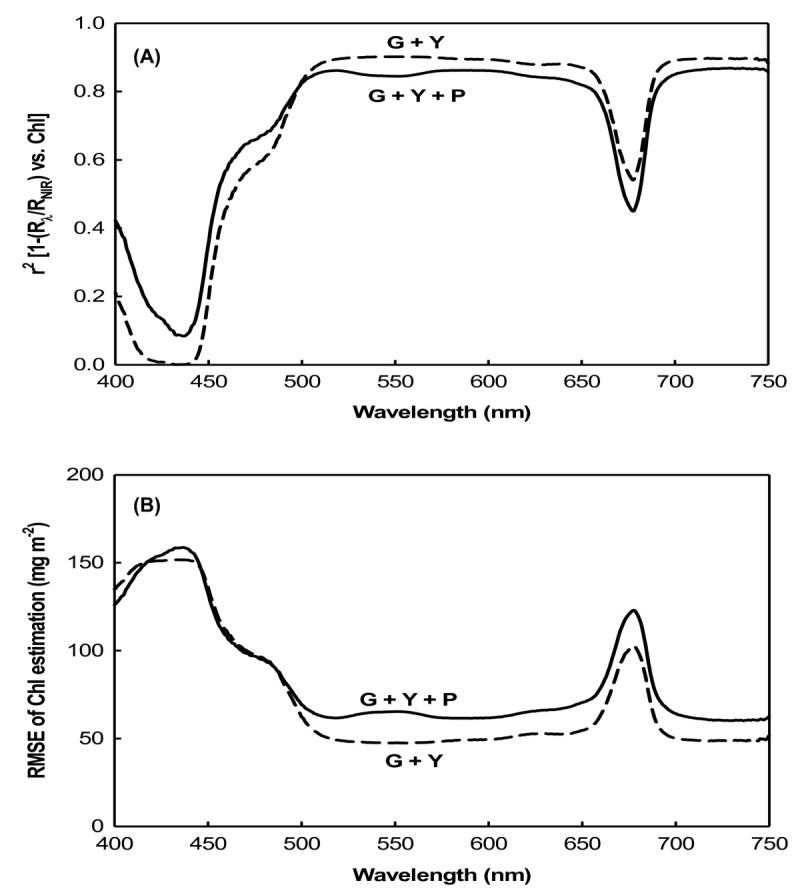
**Coefficient between [1 - (R**_**λ**_**/R**_**NIR**_**)] and the chlorophyll content and its RMSE. (A)** Coefficient between [1 - (R_λ_/R_NIR_)] and the chlorophyll content; **(B)** root mean square error (RMSE) of the chlorophyll estimation by R^-1^.

To eliminate the effect of anthocyanins on the reflectance in the green spectral range, we developed a new index, [(R_λ_)^-1^ - (R_NIR_)^-1^] × R_λ_ = 1 - (R_λ_/R_NIR_) = (R_NIR_ - R_λ_)/R_NIR_, derived from [(R_λ_)^-1^ - (R_NIR_)^-1^] and R_λ_, by just changing multiplying R_NIR_ in denominato of Gileson’s model (2006), [(R_λ_)^-1^ - (R_NIR_)^-1^] × R_NIR_ = (R_λ_/R_NIR_) - 1 = (R_NIR_ - R_λ_)/R_λ_, with multiplying R_λ_ in the new equation. The expectation for the new model is that (R_NIR_ - R_λ_)/R_NIR_, not only adjusts the difference for leaf structure, position, angle and photosynthetic pigments, but also narrows down the reciprocal spectral difference between the anthocyanin-free leaves (the less amount of anthocyanins) and the anthocyanins-containing leaves (the most amount of anthocyanins), and therefore maximally decreases the influence of anthocyanins on the chlorophyll estimation, whereas the old model (R_NIR_ - R_λ_)/R_λ_ broaden the difference and therefore maximally increases the influence. In the spectral ranges of 500 ~ 650 and 700 ~ 750 nm, the new function 1 - (R_λ_/R_NIR_) vs. chlorophyll was linear for green and yellow (G + Y) leaves (Figure 4A). As with the indices above, for this index *r*^2^ = 0.9, and the RMSE was still < 50 mg m^-2^ (Figure 4B). Unlike the previous indices, when this index was applied to the full set of leaves (G + Y + P), including the anthocyanin-containing purple leaves, there was no significant decrease in *r*^2^ in the green region of the spectrum at 500 ~ 600 nm. The present RMSE also remained low at < 70 mg m^-2^.

**Figure 5:**
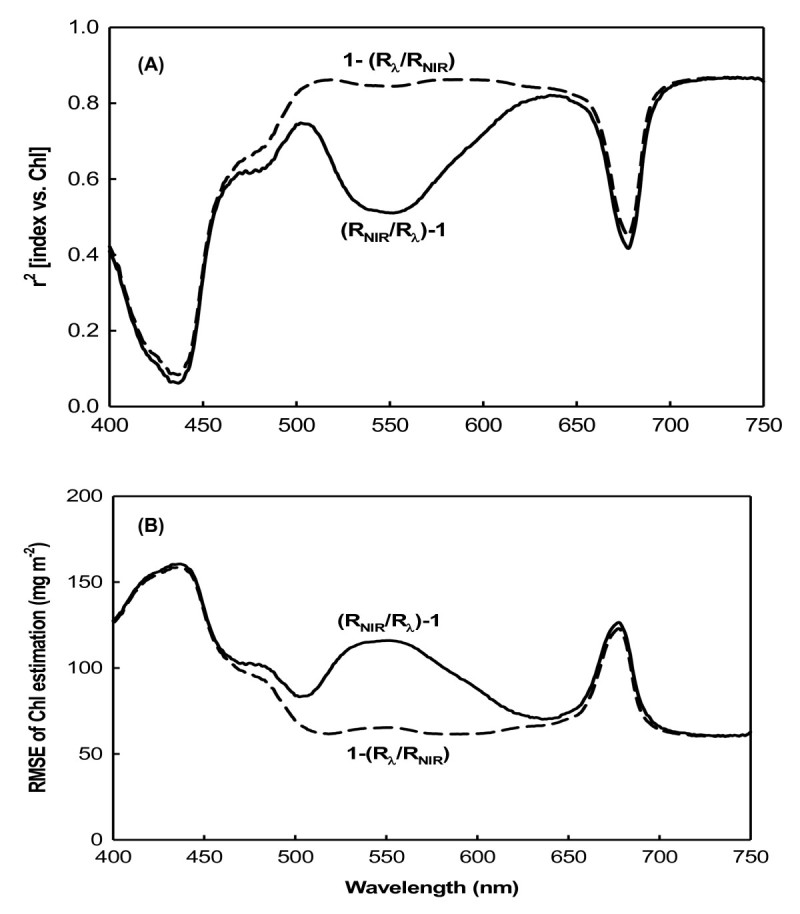
**Coefficients of [(R**_**NIR**_**/R**_**λ**_**) - 1] and [1 - (R**_**λ**_**/R**_**NIR**_**)] with the chlorophyll content of all leaves and their RMSEs. (A)** Coefficients of [(R_NIR_/R_λ_) - 1] and [1 - (R_λ_/R_NIR_)] with the chlorophyll content of all leaves (G+Y+P); **(B)** root mean square error (RMSE) of the chlorophyll estimation by R^-1^.

The main difference in the behavior of these two indices, (R_NIR_/R_λ_) - 1 (Figure 3) and 1 - (R_λ_/R_NIR_) (Figure 4), was in the green range (500 ~ 600 nm) for anthocyanin-containing leaves (Figure 5). The coefficient of (R_NIR_/R_λ_) - 1 vs. the chlorophyll content exhibited a notable decrease down to around 0.55 in the green range (Figure 5A), and the RMSE of the chlorophyll estimation using the index (R_NIR_/R_λ_) - 1 rose to 110 mg m^-2^ in the green range (Figure 5B). In contrast, the *r*^2^ of 1 - (R_λ_/R_NIR_) vs. the chlorophyll content remained around 0.8 ~ 0.9 in the green range, and the RMSE of the chlorophyll estimation by the index 1 - (R_λ_/R_NIR_) was also still minimal, remaining at < 70 mg m^-2^.

### Correlations between the predicted and actual measured chlorophyll contents

**Figure 6:**
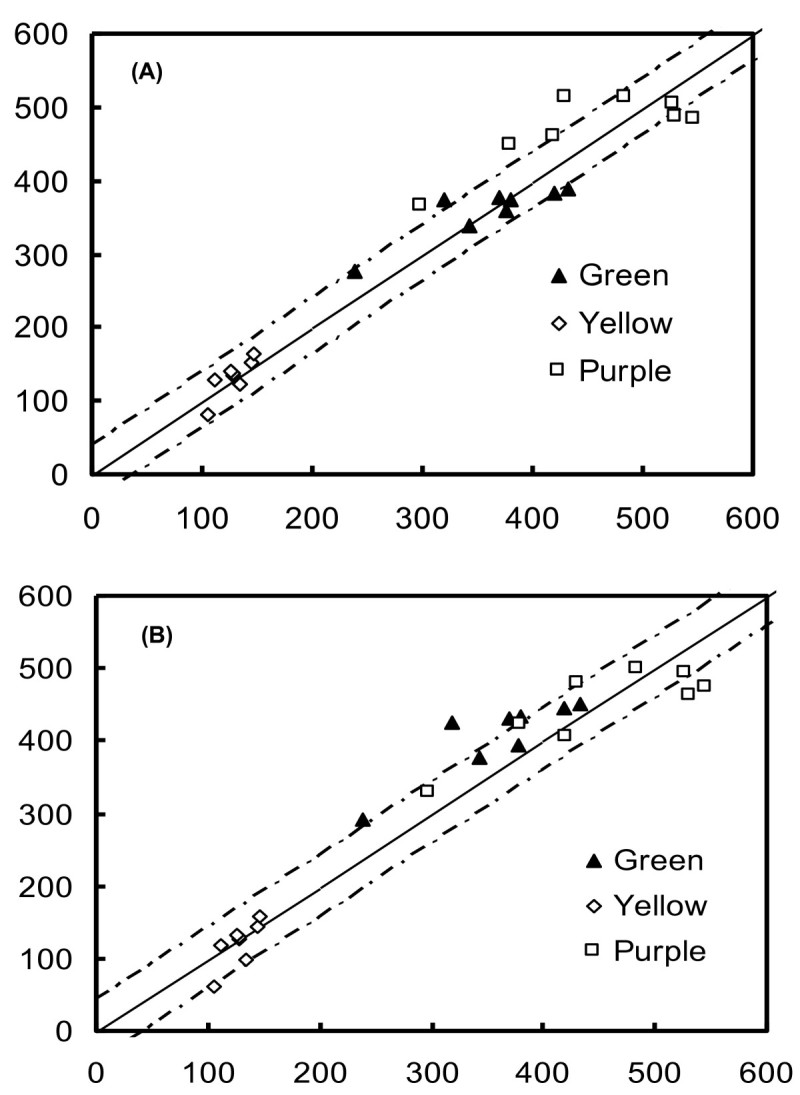
**Validation results for the index, [1 - (R**_**λ**_**/R**_**NIR**_**)], calculated using R**_**550**_ **(A) and R**_**705**_**(B) spectra from independent datasets.** The coefficients between the predicted and actual measured chlorophyll contents were *r*^2^ = 0.94 **(A)** and 0.93 **(B)**. The solid line represents the equation *Chl*_*pred*_= *Chl*_*meas*_, and the dotted lines represent the root mean square error of the chlorophyll prediction.

To validate the proposed model, the new index, 1 - (R_λ_/R_NIR_), was used to estimate the chlorophyll content in independent datasets with widely varying pigment contents (Table 1). The chlorophyll content was predicted using the reflectance data from the validation datasets, the predictions were compared to actual measured chlorophyll values, and the RMSE of the chlorophyll prediction was determined. Correlations between the predicted and measured chlorophyll contents were *r*^2^ = 0.94 for 1 - (R_550_/R_NIR_) (Figure 6A), and *r*^2^ = 0.93 for 1 - (R_705_/R_NIR_) (Figure 6B). The RMSEs of the chlorophyll predictions for R_550_ and R_705_ were < 38 and < 43 mg m^-2^, respectively.

**Figure 7:**
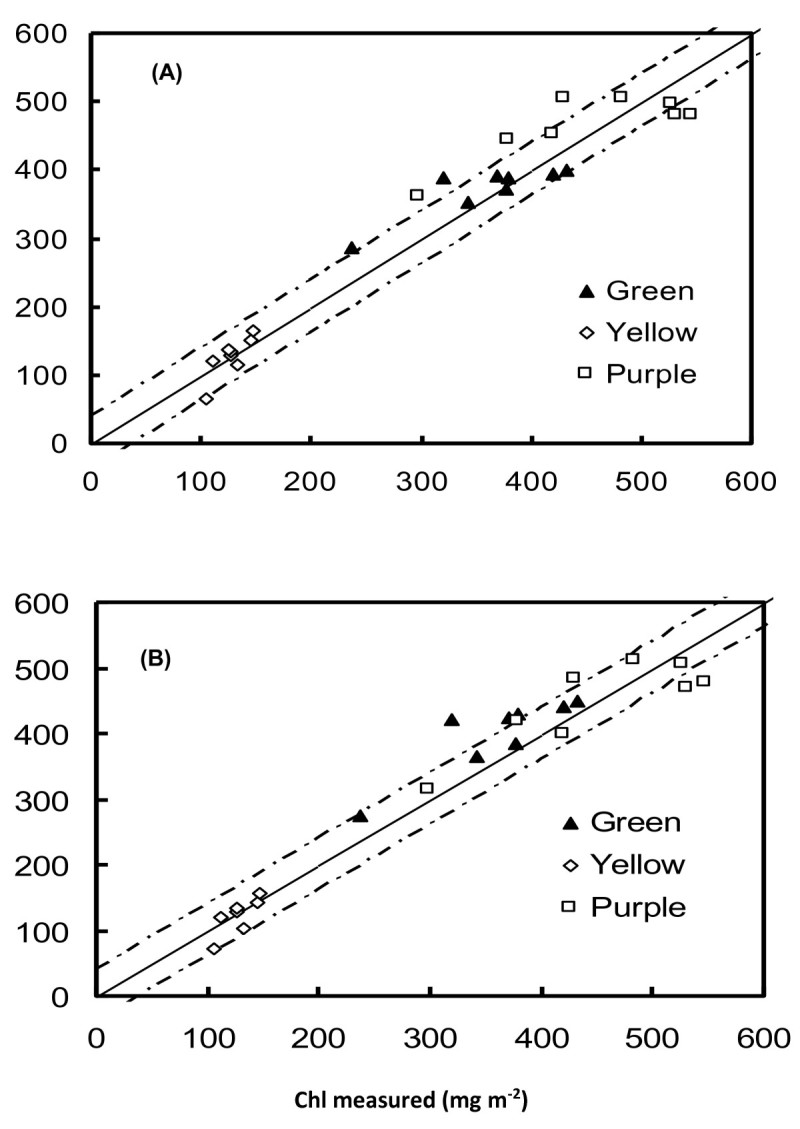
**Validation results for the index, 1 - (R**_**λ**_**/R**_**NIR**_**), calculated using broad-band spectra R**_**550~590**_**(A) and R**_**700~740**_**(B) from independent datasets.** The coefficients between the predicted and actual measured chlorophyll contents were *r*^2^ = 0.94 **(A)** and 0.94 **(B)**.

Rather than the usual narrow band channel, reflectances in the wide band ranges of 550 ~ 590 and 700 ~ 740 nm can also be used as the terms in the index, 1 - (R_λ_/R_NIR_). For the 550 ~ 590- and 700 ~ 740-nm ranges, correlations between the predicted and measured chlorophyll contents were *r*^2^ = 0.94 for both 1 - (R_550~590_/R_NIR_) (Figure 7A) and 1 - (R_700~740_/R_NIR_) (Figure 7B), with RMSEs of < 38.5 and 38.9 mg cm^-2^, respectively.

## Discussion

It was previously shown that the blue and red spectral ranges, the main absorption bands of chlorophyll, are only sensitive to low chlorophyll levels, but at moderate to high levels, they become insensitive (Gitelson and Merzlyak, 1994,1996,1997). In these spectral ranges, reflectance is only sensitive to total chlorophyll levels at 90 mg m^-2^ (Gitelson et al., 2003). Such low levels of chlorophyll are found in highly stressed or senescent leaves. The three sweet potato varieties used in this study grew well under greenhouse conditions, and the lowest chlorophyll content recorded was 99 mg m^-2^ in yellow leaves (Table 1). In both the model development and validation datasets, chlorophyll contents were not low. Therefore, the blue and red spectral reflectances for sweet potato leaves should be insensitive to chlorophyll content. For the green and yellow (G + Y) sweet potato leaves, R_λ_ values in the visible blue (400 ~ 500 nm) and visible red (660 ~ 690) ranges showed the lowest correlations with chlorophyll content, but R_λ_ values showed maximum sensitivity to the chlorophyll content in the green (500 ~ 600 nm) and red edge (around 700 nm) ranges (Figure 2A). This was due to the strong absorption by chlorophyll in the blue (400 ~ 500 nm) and red (660 ~ 690 nm) spectral ranges, but weak absorption in the green (500 ~ 600 nm) and red edge areas (around 700 nm). Therefore, reflectance in the green and red edge ranges can be used as more-precise measurements for developing leaf chlorophyll content estimation algorithms (Lichtenthaler et al., 1996; Gamon and Surfus, 1999; Gitelson et al., 2002). For green and yellow (G + Y) leaves, three indices derived from the reciprocal reflectance, [(R_λ_)^-1^ - (R_NIR_)^-1^], [(R_NIR_/R_λ_) - 1], and [1 - (R_λ_/R_NIR_)], displayed strong correlation with the total chlorophyll content in the green and red edge spectral ranges (Figures 3A, B, 4A).

The relationship between the spectral reflectance and chlorophyll content may be influenced by the presence of other pigments. Curran et al. (1991) found that the red edge was significantly shifted to longer wavelengths by amaranthin, a red pigment found in *Amaranthus tricolor* leaves. In anthocyanin-containing leaves, both chlorophyll and anthocyanin contribute to absorption and reflectance in the green spectral range. The development of a nondestructive method for estimating chlorophyll content is complicated by the interference of absorption by anthocyanins in leaves and fruits which contain them (Gitelson et al., 2001). In anthocyanin-free leaves and fruits, reflectances at the green (550 nm) and red edges (around 700 nm) were closely correlated with a wide range of chlorophyll contents (Gitelson et al., 2003). In leaves and fruits containing anthocyanins, a strong correlation was observed between the chlorophyll content and R_550_, while R_700_ was lost due to anthocyanin absorption at 550 nm causing the reflectance to be significantly lower than at 700 nm. This meant that spectral bands at around 550 nm were seen as unsuitable for nondestructive chlorophyll estimation in anthocyanin-containing leaves (Gitelson et al., 2001). In this study, only purple leaves contained anthocyanins (Table 1); therefore, correlations between the old derived indices of [(R_λ_)^-1^ - (R_NIR_)^-1^] and [(R_NIR_/R_λ_) - 1] and chlorophyll content were relatively weak in the green spectral range when the dataset included purple leaves. The RMSE of chlorophyll estimates using R^-1^ in the green spectral range was dramatically increased by the inclusion of anthocyanin-containing leaves. Independent of the presence of anthocyanins, R^-1^ indices were found to be highly sensitive to the total chlorophyll content in the red edge spectral range (Figures 2, 3).

When developing indices for nondestructive chlorophyll estimation, spectral bands at around 550 nm are not recommended for leaves which contain anthocyanins (Gitelson et al., 2003). Hence, we attempted to determine whether spectral reflectance in the green range could be used as a sensitive term in developing algorithms for estimating the chlorophyll content of leaves containing anthocyanins. The new index, [1 - (R_λ_/R_NIR_)], was therefore developed to eliminate the effect of anthocyanins on reflectance in the green spectral range. Using this new index to predict the chlorophyll content, no sharp decrease in *r*^2^ or increase in RMSE was observed following the inclusion of purple leaves in the dataset (Figures 4, 5). This means that the green range reflectance becomes a usable term for estimating the chlorophyll content in anthocyanin-containing leaves.

**Figure 8:**
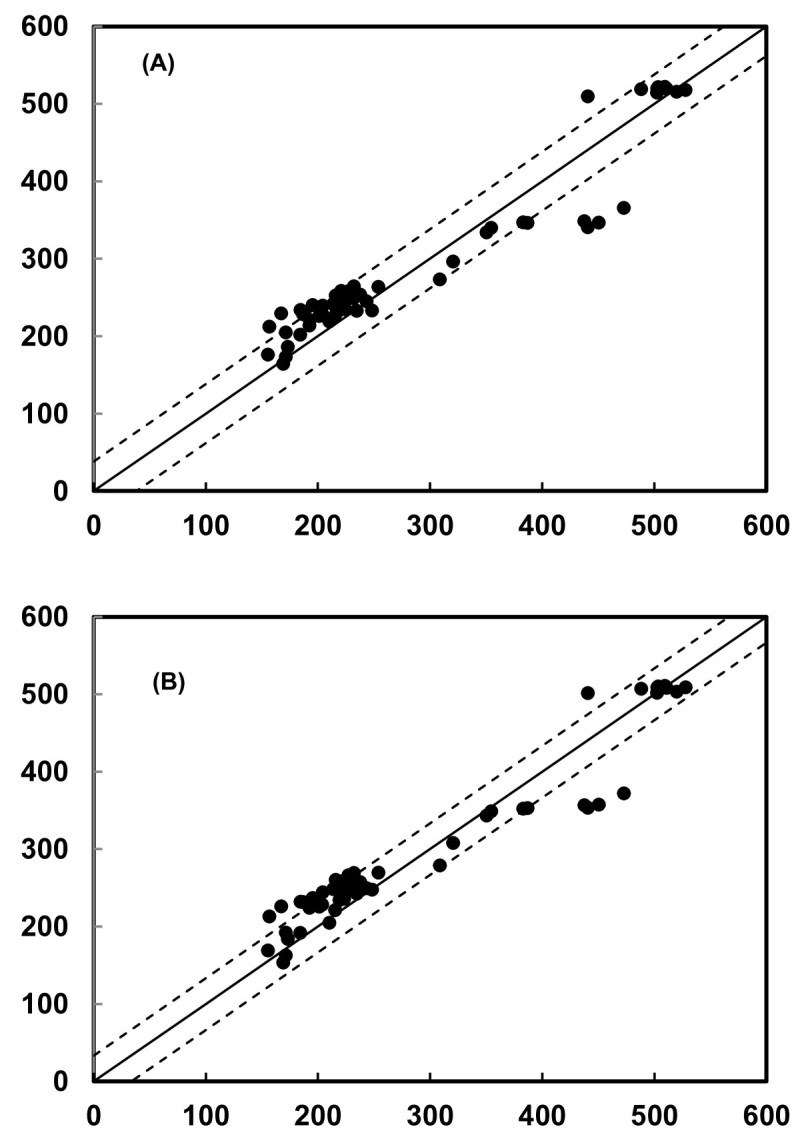
**Validation results for the index, [1 - (R**_**λ**_**/R**_**NIR**_**)], calculated using R**_**550**_**(A) and broad-band spectra R**_**550-590**_**(B) from rice data.** The coefficients between the predicted and actual measured chlorophyll contents were *r*^2^ = 0.91 **(A)** and 0.92 **(B)**. The solid line represents the equation *Chl*_*pred*_ = *Chl*_*meas*_, and the dotted lines represent the root mean square error of the chlorophyll prediction.

With validation of the indices, [1 - (R_550_/R_NIR_)] and [1 - (R_705_/R_NIR_)], they are shown to be accurate predictors of the chlorophyll content of leaves containing anthocyanins (Figure 6). To significantly increase the sensitivity and signal-to-noise ratio and decrease the cost of reflectometers, the use of wide-band filters is allowed in reflectometers (Gitelson et al., 2003). Reflectances in the wide band ranges of 550 ~ 590 and 700 ~ 740 nm were used as parameters in the index, 1 - (R_λ_/R_NIR_): 1 - (R_550~590_/R_NIR_) and 1 - (R_700~740_/R_NIR_). Validation demonstrated that the chlorophyll content could be accurately estimated for leaves which contained anthocyanins (Figure 7). Therefore, independent of the presence of anthocyanins, spectral bands in the green or red edge ranges are sufficient for nondestructive chlorophyll estimation when used as parameters in the index, 1 – (R_λ_/R_NIR_). This new index, 1 - (R_λ_/R_NIR_), and old index, (R_NIR_/R_λ_) - 1, have been applied to nondestructively assess the chlorophyll content of yellow, green and purple rice cultivars; the coefficients between the predicted and actual measured chlorophyll contents were *r*^2^ = 0.91 and 0.92, respectively (Figure 8). Therefore, it is expected that the present new index could be comprehensively used to nondestructively estimate the plant leaves containing anthocyanins with high *r*^2^ and low RMSE, while other indices in the literature are not.

## Conclusions

In previous studies, spectral bands in green were determined to be unsuitable for chlorophyll nondestructive estimation with leaves containing anthocyanins (Gitelson et al., 2006; Merzlyak et al., 2003). In the present study, we developed a new index to eliminate the effect of anthocyanins on the reflectance in the green range when estimating chlorophyll contents of leaves containing anthocyanins. We concluded that the index, [1 - (R_λ_/R_NIR_)], used with reflectance in the green and red edge ranges, is sufficient for nondestructive chlorophyll estimation in spite of the presence or absence of anthocyanins. Furthermore, the new index is more comprehensively applicable to nondestructively estimate the chlorophyll contents in any kind of leaves and fruits with any level of anthocyanins.

## Electronic supplementary material

Additional file 1:**Multilingual abstracts in the six official working languages of the United Nations.**(DOC 24 KB)

Below are the links to the authors’ original submitted files for images.

Authors’ original file for figure 1

Authors’ original file for figure 2

Authors’ original file for figure 3

Authors’ original file for figure 4

Authors’ original file for figure 5

Authors’ original file for figure 6

Authors’ original file for figure 7

Authors’ original file for figure 8
