# Supplementary material for: Eliminating interference by anthocyanin in chlorophyll estimation of sweet potato (Ipomoea batatas L.) leaves
Source: Bot Stud. 2014 Jan 30;55:11. doi: 10.1186/1999-3110-55-11 (PMC5432896; doi:10.1186/1999-3110-55-11)
Supplement: Supplementary file 1 — Additional file 1:Multilingual abstracts in the six official working languages of the United Nations.(DOC 24 KB) [file 40529_2013_66_MOESM1_ESM.doc]

**中文摘要**

本文運用反射光譜發展一新模式，使能更精確地非破壞性地估算在溫室生長22天的綠、黃及紫色甘藷葉之葉綠素含量。三種甘薯葉之光合綠素含量歧異很大，但只有紫色葉含大量花青素，其他二種則少量或無。對綠與黃色甘藷葉，反射光譜倒數(R^-1^)及其衍生指標，[(R_λ_)^-1^ - (R_NIR_)^-1^] and [(R_NIR_/R_λ_) - 1]，在綠與紅光臨界區與葉綠素含量有明顯的相關性(*r*^2^=0.8~0.9)，其葉綠素估算的RMSE< 50 mg m^-2^。當含大量花青素的紫甘藷葉與前面綠與黃甘藷葉一起測驗時，前述指標與葉綠素含量在綠光臨界區之相關性就很薄弱，且其RMSE大幅增為> 110 mg m^-2^。本文新發展的指標[1 - (R_λ_/R_NIR_)]在綠光臨界區可消除花青素對葉綠素估算的扭曲效應。對含高量花青素的葉綠素估算，新指標[1 - (R_λ_/R_NIR_)]仍有很高的相關性(*r*^2^=0.8~0.9)，而其RMSE則降到最低。因此，此反射指標[1 - (R_λ_/R_NIR_)]代表一新的可有效、更精確、非破壞性估算含各不同含量花青素的所有顏色葉片之葉綠素含量，諸如紫葉水稻。

關鍵詞：甘藷葉，葉綠素，花青素，光譜反射
