# Supplementary figures and images for: Eliminating interference by anthocyanin in chlorophyll estimation of sweet potato (Ipomoea batatas L.) leaves
Source: Bot Stud. 2014 Jan 30;55:11. doi: 10.1186/1999-3110-55-11 (PMC5432896; doi:10.1186/1999-3110-55-11)

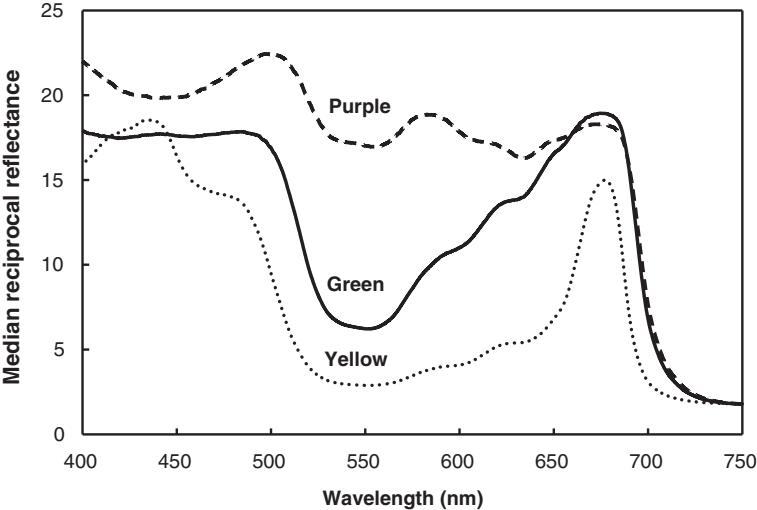

Supplement: Supplementary file 2 — Authors’ original file for figure 1 [file 40529_2013_66_MOESM2_ESM.pdf]

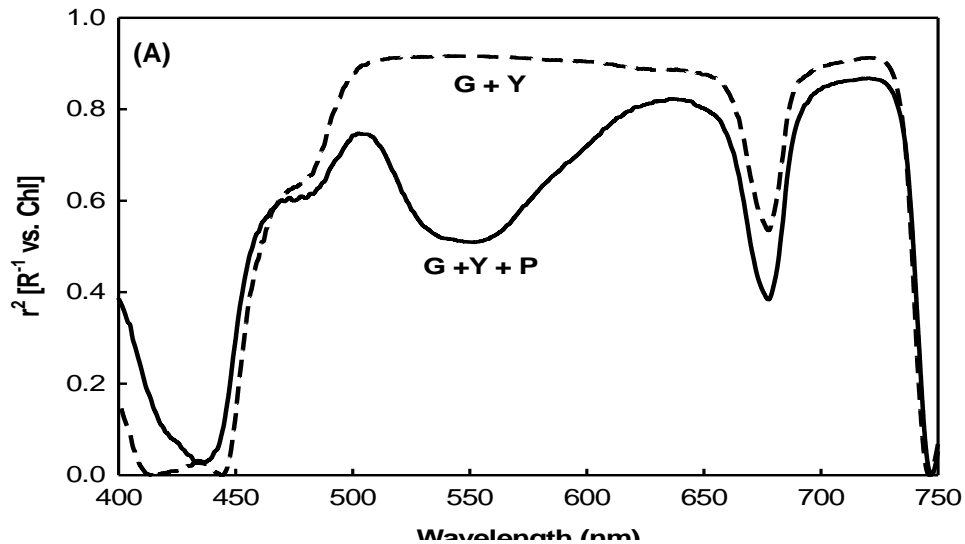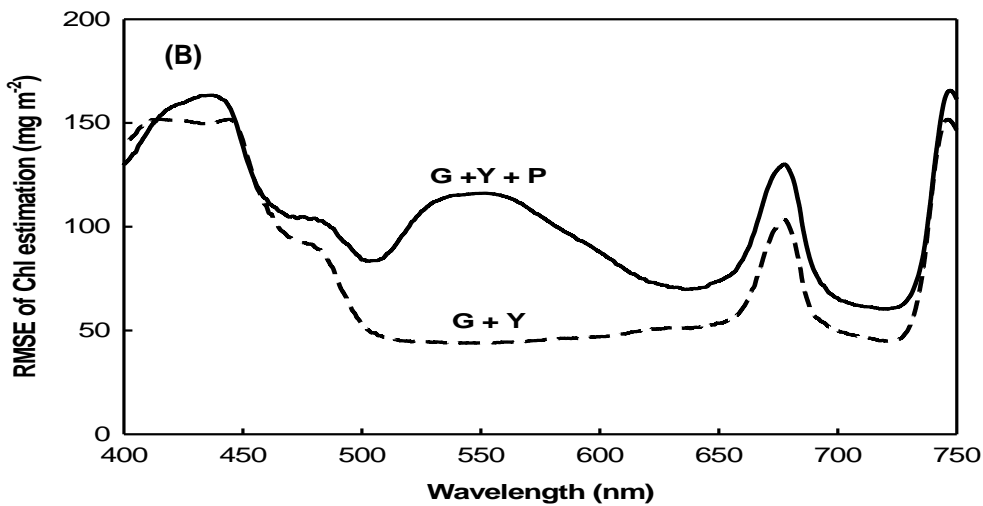

Supplement: Supplementary file 3 — Authors’ original file for figure 2 [file 40529_2013_66_MOESM3_ESM.pdf]

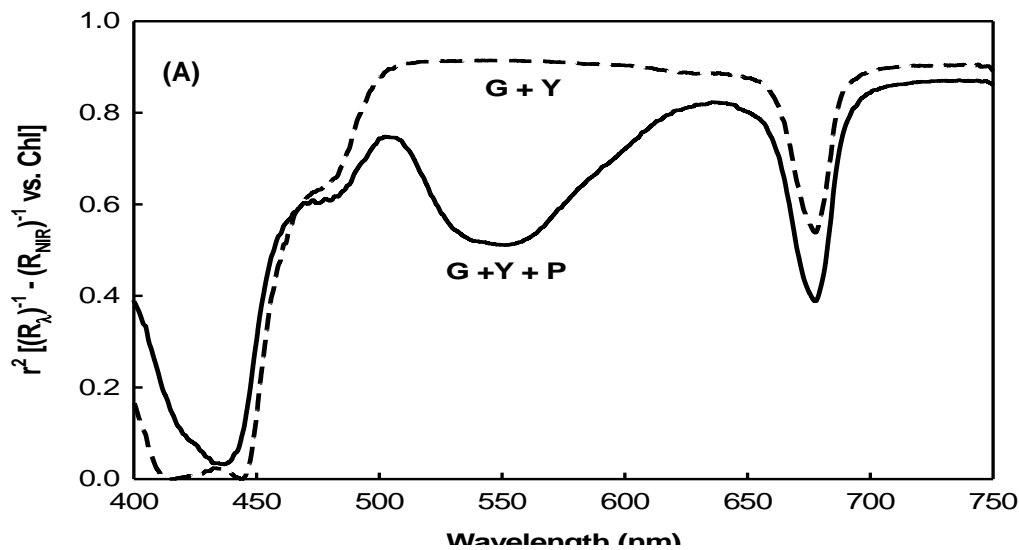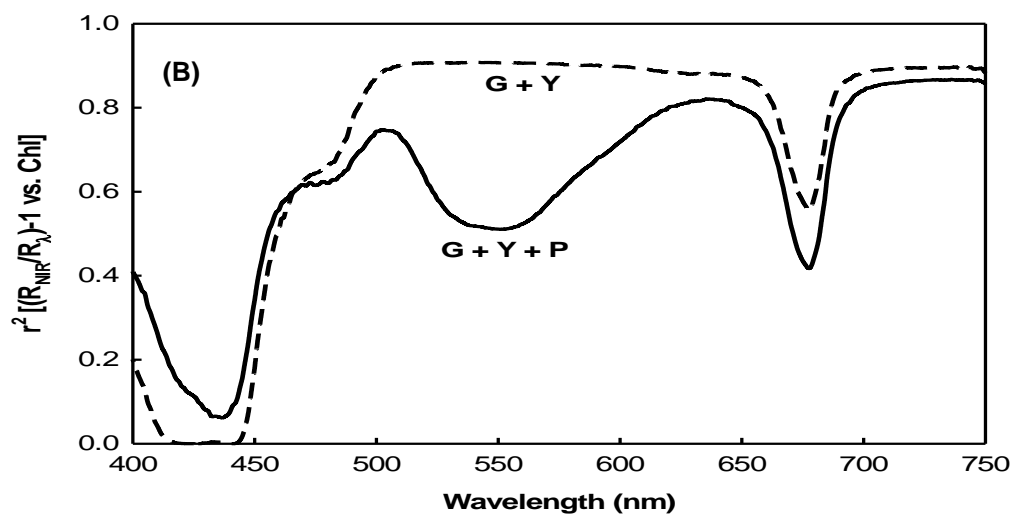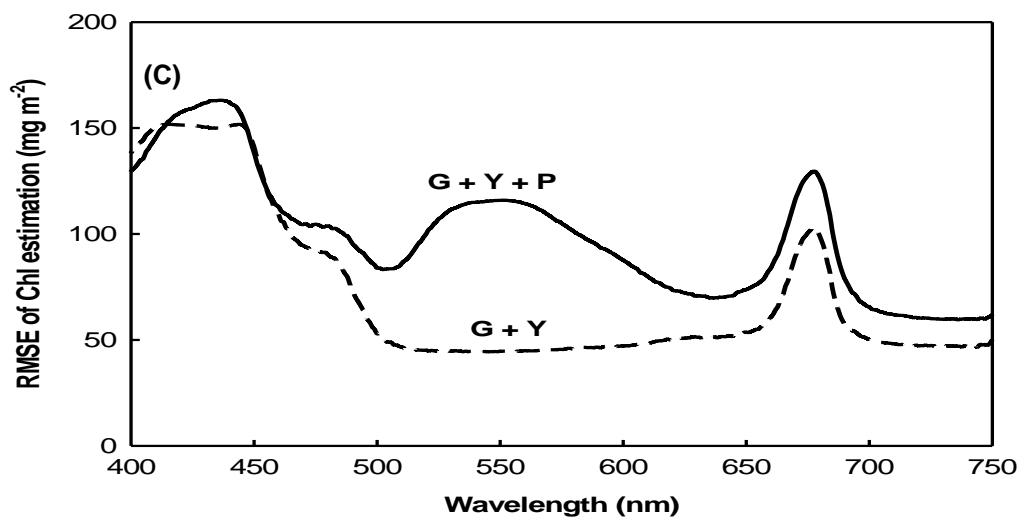

Supplement: Supplementary file 4 — Authors’ original file for figure 3 [file 40529_2013_66_MOESM4_ESM.pdf]

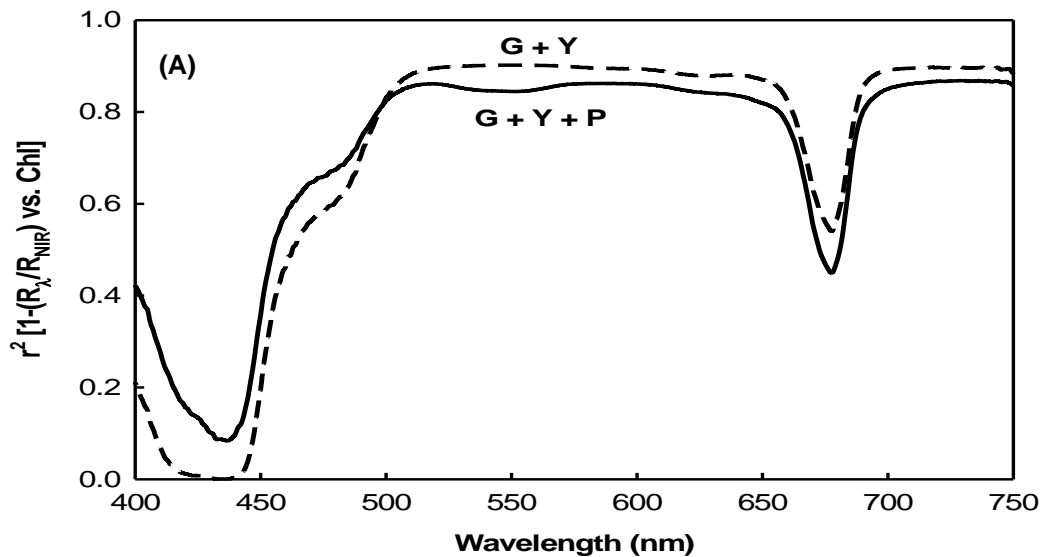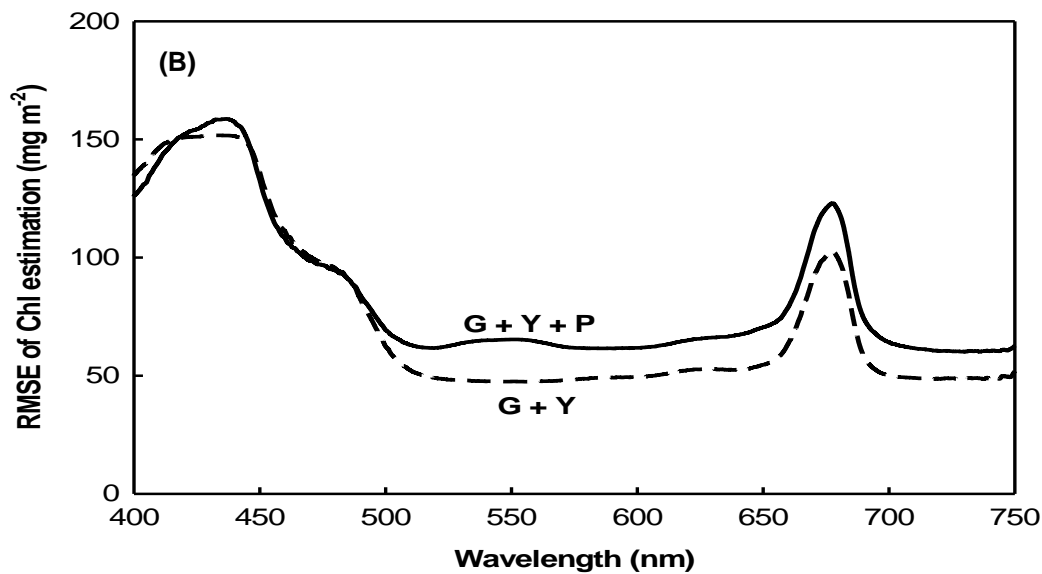

Supplement: Supplementary file 5 — Authors’ original file for figure 4 [file 40529_2013_66_MOESM5_ESM.pdf]

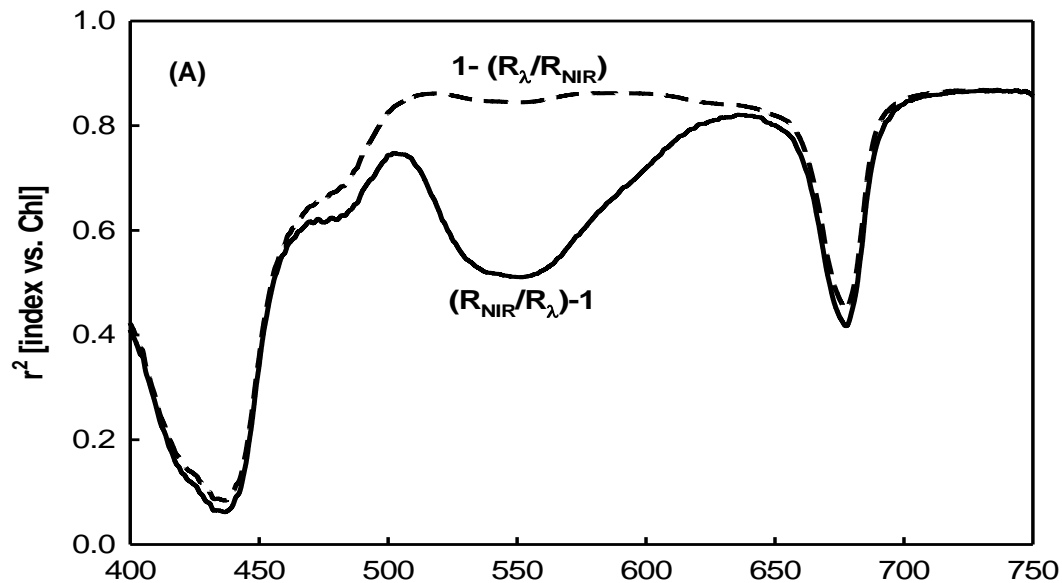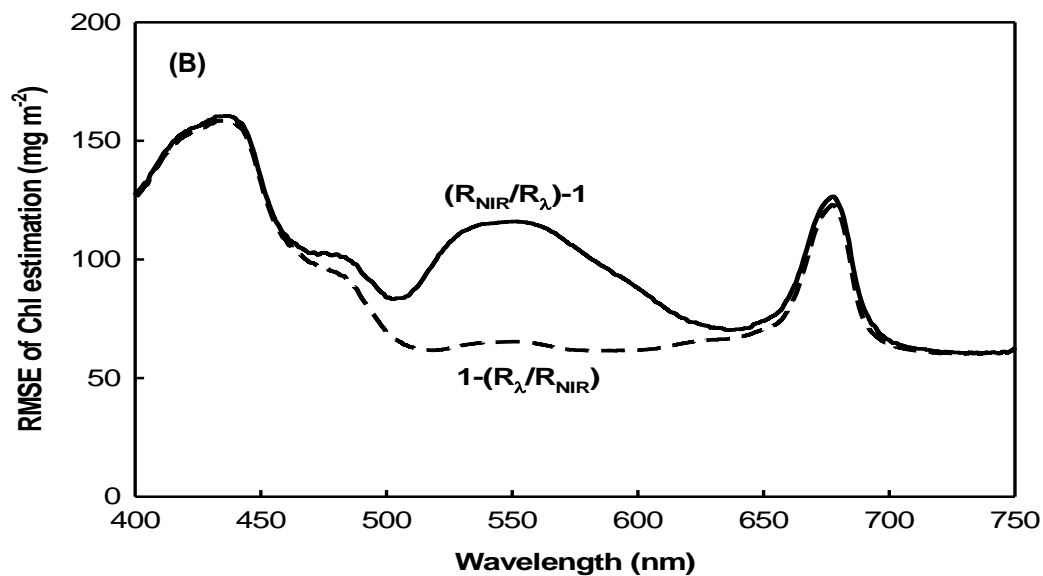

Supplement: Supplementary file 6 — Authors’ original file for figure 5 [file 40529_2013_66_MOESM6_ESM.pdf]

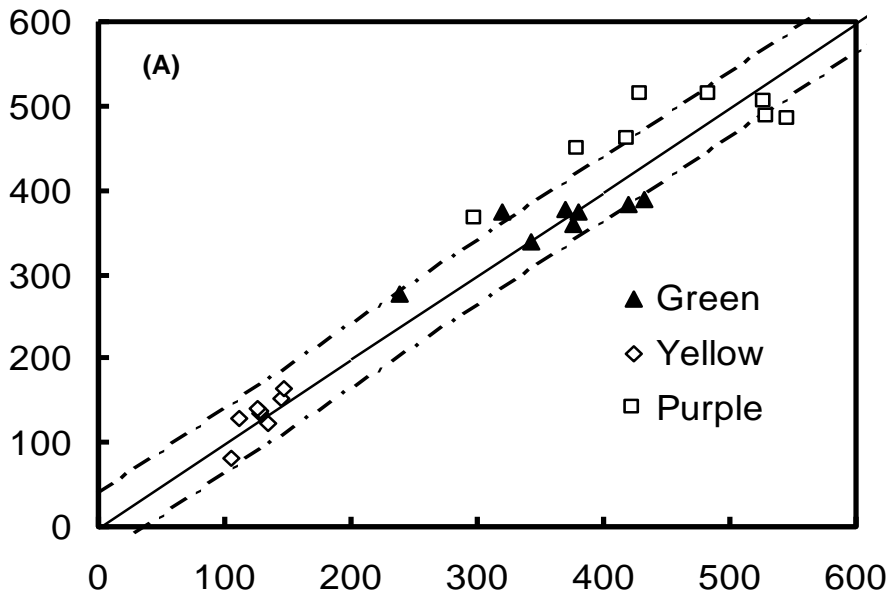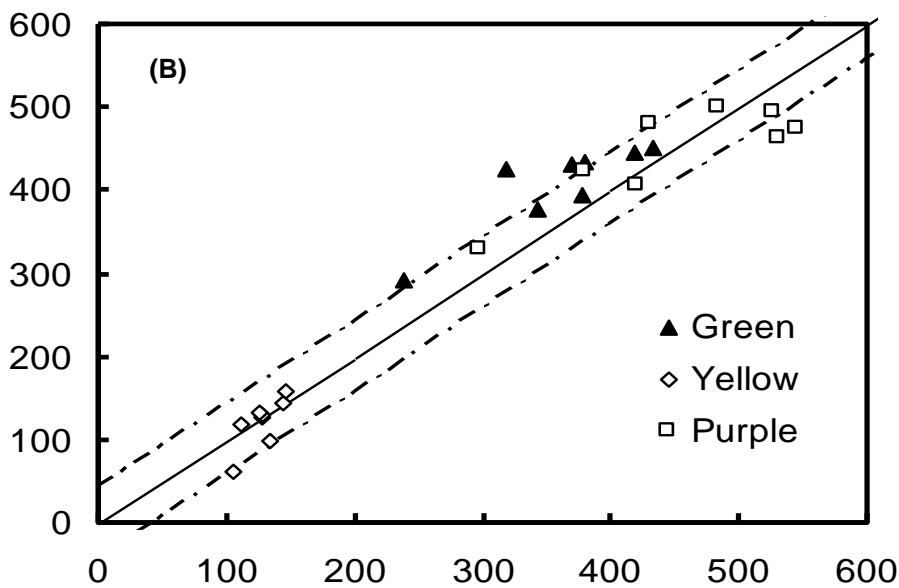

Supplement: Supplementary file 7 — Authors’ original file for figure 6 [file 40529_2013_66_MOESM7_ESM.pdf]

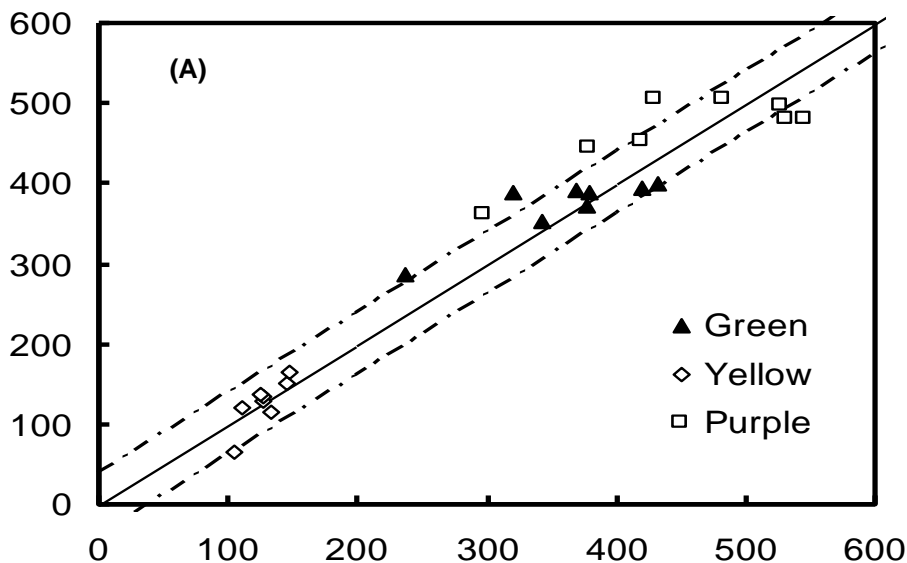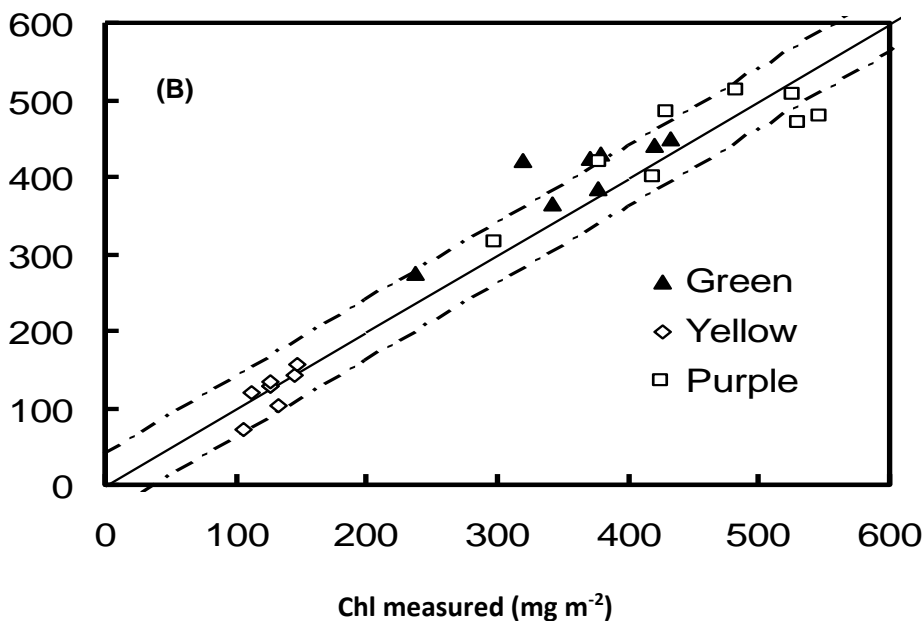

Supplement: Supplementary file 8 — Authors’ original file for figure 7 [file 40529_2013_66_MOESM8_ESM.pdf]

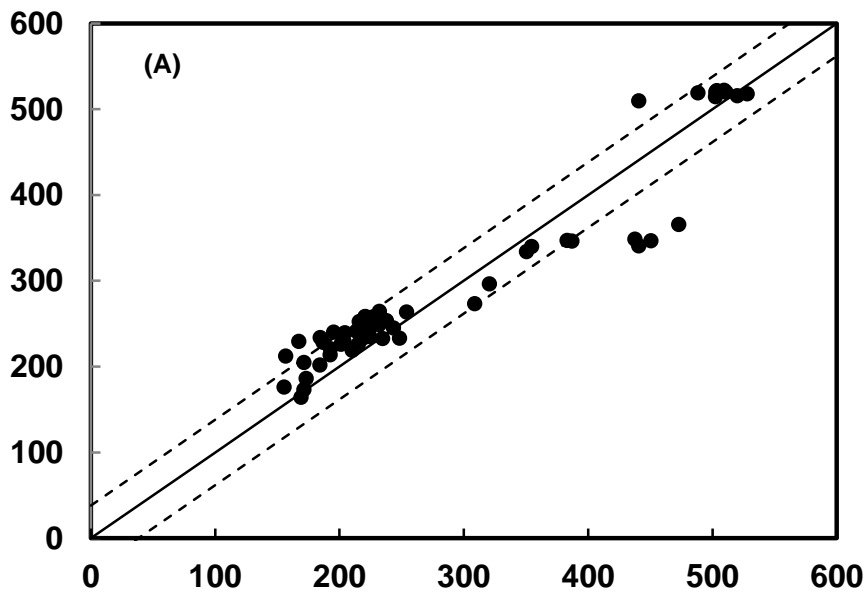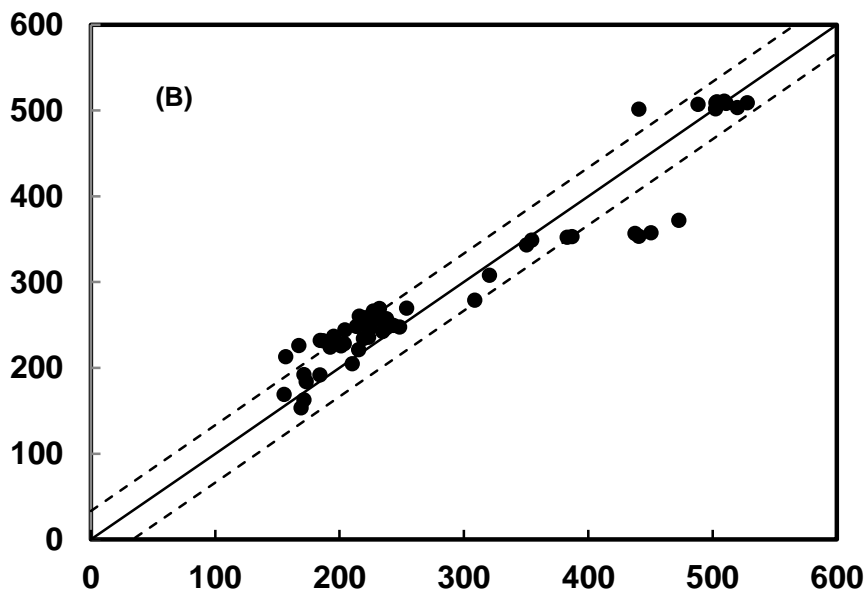

Supplement: Supplementary file 9 — Authors’ original file for figure 8 [file 40529_2013_66_MOESM9_ESM.pdf]
